# Cucurbitacin-I (JSI-124) activates the JNK/c-Jun signaling pathway independent of apoptosis and cell cycle arrest in B Leukemic Cells

**DOI:** 10.1186/1471-2407-11-268

**Published:** 2011-06-24

**Authors:** Ganchimeg Ishdorj, James B Johnston, Spencer B Gibson

**Affiliations:** 1 Manitoba Institute of Cell Biology, Winnipeg, MB, Canada; 2 Department of Biochemistry and Medical Genetics, University of Manitoba, Winnipeg, MB, Canada; 3 Department of Internal Medicine, University of Manitoba, Winnipeg, MB, Canada

**Keywords:** JSI-124, VEGF, c-Jun, angiogenesis

## Abstract

**Background:**

Cucurbitacin-I (JSI-124) is potent inhibitor of JAK/STAT3 signaling pathway and has anti-tumor activity in a variety of cancer including B cell leukemia. However, other molecular targets of JSI-124 beyond the JAK/STAT3 pathway are not fully understood.

**Methods:**

BJAB, I-83, NALM-6 and primary CLL cells were treated with JSI-124 as indicated. Apoptosis was measured using flow cytometry for accumulation of sub-G1 phase cells (indicator of apoptosis) and Annexin V/PI staining. Cell cycle was analyzed by FACS for DNA content of G1 and G2 phases. Changes in phosphorylation and protein expression of p38, Erk1/2, JNK, c-Jun, and XIAP were detected by Western blot analysis. STAT3 and c-Jun genes were knocked out using siRNA transfection. VEGF expression was determined by mRNA and protein levels by RT-PCR and western blotting. Streptavidin Pull-Down Assay was used to determine c-Jun binding to the AP-1 DNA binding site.

**Results:**

Herein, we show that JSI-124 activates c-Jun N-terminal kinase (JNK) and increases both the expression and serine phosphorylation of c-Jun protein in the B leukemic cell lines BJAB, I-83 and NALM-6. JSI-124 also activated MAPK p38 and MAPK Erk1/2 albeit at lower levels than JNK activation. Inhibition of the JNK signaling pathway failed to effect cell cycle arrest or apoptosis induced by JSI-124 but repressed JSI-124 induced c-Jun expression in these leukemia cells. The JNK pathway activation c-Jun leads to transcriptional activation of many genes. Treatment of BJAB, I-83, and NALM-6 cells with JSI-124 lead to an increase of Vascular Endothelial Growth Factor (VEGF) at both the mRNA and protein level. Knockdown of c-Jun expression and inhibition of JNK activation significantly blocked JSI-124 induced VEGF expression. Pretreatment with recombinant VEGF reduced JSI-124 induced apoptosis.

**Conclusions:**

Taken together, our data demonstrates that JSI-124 activates the JNK signaling pathway independent of apoptosis and cell cycle arrest, leading to increased VEGF expression.

## Background

Cucurbitacin-I (JSI-124) can be found in a variety of plants that have been used for centuries as folk medicines in Asia [1-3]. However, the molecular mechanisms responsible for the various biological effects of JSI-124 have not been fully investigated. JSI-124 is a selective dual inhibitor of phospho-JAK2 and phospho-STAT3 in human breast cancer, lung cancer, neuroblastoma, and murine melanoma cell lines [4,5]. This inhibitor has been shown to exert anti-proliferative and anti-tumor activity both *in vivo *and *in vitro *[2,4,6]. More recently we have shown that JSI-124 can induce apoptosis and cell cycle arrest in B-cell leukemia cell lines and in primary chronic lymphocytic leukemia (CLL) cells [7]. It is possible that the anti-tumor effects of JSI-124 could be explained by the inhibition of the constitutively activated STAT3 signaling pathway in leukemia [7]. Independent of its effects on STAT3, JSI-124 was shown to interfere with LPA-mediated up-regulation of connective tissue growth factor (CTGF), as demonstrated in wild type and STAT3 knock-out mouse embryonic fibroblasts [8]. Thus far, these different activities of JSI-124 has not been investigated in parallel, and the specificity of the compound as an inhibitor of STAT3 signaling has not been defined in relation to the effects of the drug on other signaling pathways.

Chemotherapeutic drugs often induce a stress response in cancer cells. One of the early stress response pathways is the c-Jun N-terminal kinase (JNK) pathway [9]. JNK is a member of the mitogen activated protein kinase (MAPK) family that includes p38 and Erk1/2. The JNK pathway is activated by a variety of stimuli including UV radiation and DNA damaging agents. It has been demonstrated that this pathway could contribute to apoptosis and regulation of gene expression [9,10]. JNK regulates gene expression though phosphorylation of c-Jun and activation of the AP-1 complex [10]. These genes regulate many functions including cell survival, cell death, and angiogenesis [10].

Vascular endothelial growth factor (VEGF), known as VEGF-A, belongs to the cysteine-knot superfamily of growth factors, which is the crucial regulator of angiogenesis [11]. Angiogenesis is a complex multistep process, which can contribute to cancer progression, tumor growth and metastasis [12,13]. Although VEGF is a cytokine that regulates normal hematopoiesis, VEGF can act as an auto-and paracrine stimulator of cell survival and angiogenesis in hematological malignancies such as chronic myelogenous leukemia and chronic lymphocytic leukemia (CLL) [14,15]. VEGF expression has been related to induction of the c-Jun N-terminal kinase (JNK) signaling pathway with activation of transcription factors such as HIF-1 [16,17]. We and others have previously demonstrated that lysophosphatidic acid (LPA) increases VEGF expression involving JNK and transcription factor NF-kB in Burkitt lymphoma cell line (BJAB) and breast cancer cells [18-20]. VEGF levels are elevated in the plasma of patients with CLL, where LPA mediated protection against apoptosis in these cells through the activation of VEGF receptors [21].

In this study, we addressed the non-STAT3 effect of JSI-124 in leukemic cancer cells. Our results indicated that JSI-124 treatment induced activation of JNK signaling pathway leading to increased VEGF expression that is independent of STAT3.

## Methods

### Materials

JSI-124 was purchased from Calbiochem Inc. and dissolved in DMSO at an initial stock concentration of 10 mM. Rabbit polyclonal antibodies against phosphorylated or total c-Jun, JNK, p38, phosphor and total Erk1/2, XIAP and STAT3 antibodies, as well as siRNA for c-Jun (#6203) and Stat3 siRNA I (#6580) and control siRNA (#6201 fluorescein conjugate) were purchased from Cell Signaling Technology, VEGF antibody was purchased from ThermoScientific and Santa Cruz. Soluble super-TRAIL was purchased from ALEXIS. Annexin V-FITC and propidium iodide (PI) were acquired from Pharmingen (BD Biosciences). SP600125 and SB230580 were purchased from CalBiochem. U0126 was purchased from LC Labs, USA. All inhibitors were dissolved in DMSO. Cells were treated with the appropriate volume of DMSO for the vehicle control.

### Cells

Burkitt lymphoma cell line **-**BJAB was obtained from the American Type Culture Collection (Cedarlane Lab, Canada) and pre-acute lymphocytic leukemia cell line, NALM-6 was obtained from DSMZ (Braunschweig, Germany). Cells were cultured and frozen down from the first passage for future use. Cells were characterized by the cell bank by staining with: CD3 -, CD10 +, CD19 +, CD37 -, cyCD79a +, CD80 -, CD138 +, HLA-DR +, sm/cyIgG -, cyIgM +, smIgM -, sm/cykappa -, sm/cylambda. I-83 was a kind gift from Dr. Panasci (McGill University). All cell lines were grown in RPMI 1640 medium (HyClone-ThermoScientific) containing 10% FCS (HyClone ThermoScientific) in a humidified atmosphere (37°C; 5% CO2). All growing cells were routinely tested for mycoplasma every 3 months. Primary B-CLL cells were isolated and maintained as previously described [22].

### Cell lysate and Immunoblot Analysis

Lysates were prepared from BJAB, I-83, NALM-6 cells stimulated with JSI-124 with lysis buffer (50 mM TRIS pH = 7.4, 150 mM NaCl, 1% NP40, 0.5% sodium deoxycholate, 0.1% SDS, with protease and phosphatase inhibitors). The cell debris was removed by centrifugation at 13,000 × g for 5 min at 4°C, and the protein concentration was determined using the Bradford assay. After SDS-PAGE, the proteins were transferred to nitrocellulose membrane. The membranes were blocked with 5% milk in TBS-Tween 20 solution for 1 h, followed by overnight incubation with appropriate primary antibody at 4°C and finally incubated for 1 h with secondary antibody conjugated with horseradish peroxidase (Bio-Rad). Detection was by ECL-Western Lightning Chemiluminescence reagent (Amersham Pharmacia). The blots were stripped with Western-Re-Probe reagent (CalBiochem) and re-probed for β-actin to normalize for protein loading in each lane.

### Transfection of BJAB, I-83 cells with siRNA

2.5 × 10^6 ^cells were used for electrophoresis (Nucleofactor l Device-Lonza) with Cell line Nucleofection Kit-T-program-O17 or C05 (Lonza) according to the manufacturer's instruction. siRNA-100 nM against c-Jun or STAT3 or non-targeting control were used. After 48 hours of transfection the cells were analyzed by FACS for cell cycle analysis or cell lysate was prepared for immunoblotting as described above. Transfection efficiency was confirmed by transfecting 2 ug pmax-GFP or fluorescein conjugated control siRNA using the same condition described and then visualizing the cells using by fluorescence microscopy.

### Real-time PCR

Real-time PCR was used to assess mRNA level of VEGF in cells that had been treated with JSI-124 for various times. Total RNA was isolated using the RNeasy RNA Isolation Kit (Qiagen) and 2 μg of total cellular RNA was used as a template for reverse transcription-PCR (RT-PCR) with random hexamers (Invitrogen). cDNA was then used as a template for real-time PCR using pre-designed primer sets and SYBR Green-PCR Master Mix (BIO-RAD) according to the manufacturer's instructions. The following primers were used for the real time-PCR reaction: mRNA-VEGF-F-5'-ACAACAAATGTGAATGCAGACC-3' R-5'-TTTGCAGGAACATTTACACGTC-3'. Primers to the housekeeping gene GAPDH were used to standardize results F-5' TCCATGACAACTTTGGTATCGTGG3', R-5'GACGCCTGCTTCACCACCTTCT3'.

### Streptavidin Pull-Down Assay

Nuclear extract from the cells treated with JSI-124 or vehicle was pre-cleared with Streptavidin Agarose beads for 30 minutes. 500 ug of pre-cleared extract was added with 50 ng/ul Poly d(I-C), 100 nM biotin labelled DNA probe (The probe sequence is as follows: AP1 5'-CGCTTGATGACTCAGCCGG AA-3') or control probes (mAP1 5'-CGCTTGATGACTTGGCCGGAA-3') in binding buffer (50 mM Tris pH 7.5, 250 mM KCl, 5 mM DTT) and incubate at room temperature for 30 min. The probes were pulled down using 50 ul Streptavidin-agarose. The complex was incubated for 30 minutes, washed 3 times with PBS and Western blotted for transcription factors expression. Nuclear extract were used as a positive control.

### Cell death detection and Annexin V/7-AAD Staining

The cell death assay was performed as described by Ishdorj et al [12]. Briefly, cells (5 × 10^6^) were collected in 5 ml tubes, centrifuged, and resuspended in 1× binding buffer (10 mM HEPES/NaOH, pH 7.4, 140 mM NaCl, 2.5 mM CaCl_2_) supplemented with annexin V-fluorescein isothiocyanate (BD PharMingen, Mississauga, ON) and 2.5 μg of 7-AAD or PI (Molecular Probes, Eugene, OR). After 15 min of incubation at room temperature in the dark, an excess of 1× binding buffer was added to a final volume of 500 μl. The cells were then analyzed for FACS with Calibur flow cytometer.

### Cell Cycle Analysis

Cells were fixed at least 1 hour with 70% (w/v) ice-cold ethanol at 4°C. Cells were washed with PBS and resuspended in 1 mL of PBS containing 50 μg/mL PI and 500 U/mL RNase A. Following incubation for 15 min in the dark at room temperature, cells were analyzed by flow cytometer using the CellQuest software (Becton Dickinson, San Jose, CA, USA). The PI fluorescence signal at FL2A peak versus the count was used to discriminate G2-M cells from G0-G1 doublets.

### SupperArray Analysis

JSI-124 or DMSO treated BJAB cells were used for SupperArray analysis with 96 wells Human Signal Transduction PathwayFinder™ RT^2 ^Profiler™ (SABiosciences, QIAGEN) according to the manufacturer's instruction. Briefly, total RNA was isolated using the RNeasy RNA Isolation Kit (Qiagen) and 2 μg of total RNA was used for RT^2 ^First Strand Kit (SABiosciences, QIAGEN). The PCR Array was performed in 96 wells and included five housekeeping genes. Controls are included on each array for genomic DNA contamination, RNA quality and general PCR performance. Data was analysis by RT^2 ^Profiler™ PCR Array Data Analysis Software.

### Statistical Analysis

All experiments were repeated at least three times and each experiment was performed at least in duplicate. The data were expressed as means ± SE (standard error). Statistical analysis was performed by using Student's *t *test. The criteria for statistical significance was *p *< 0.05.

## Results

### JSI-124 induced activation of c-Jun N-terminal kinase (JNK) and c-Jun in B leukemic cell lines

**Figure 1:**
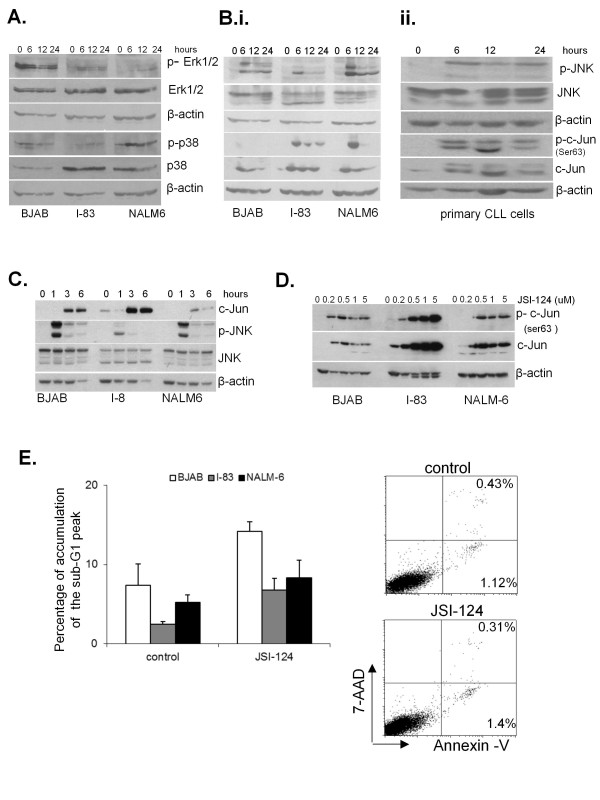
**JSI-124 induces phosphorylation of c-Jun in B-leukemia cells**. **A, **and** B**. I-83, BJAB, NALM-6 cells were treated with 1 μM JSI-124 for a 6 and 24 hour time course. Cell lysates were used for immunoblotting for the presence of p-JNK/JNK, p-p38/p38, p-p42/44 or p42/44 and p-c-Jun/c-Jun. Experiments were done at least in triplicate for all cells and for primary CLL cells. Representative original data are shown in Fig.1. **C**. All three cell lines were treated with 1 μM JSI-124 for 6 hours. **D**. Cells were treated with various doses of JSI-124 for 6 hours. Phospho-and total c-Jun levels were assessed by immunoblotting. **E**. BJAB, I-83, and NALM-6 cells were treated with 0.2 μM concentration of JSI-124 for 24 hour. Apoptosis was measured using flow cytometric analysis for accumulation of sub-G1 phase (upper panel) or Annexin V/7AAD staining. Data represents 3 independent experiments. Standard error was determined on the basis of three independent experiments.

MAPKs are serine/threonine-specific protein kinases that respond to extracellular stimuli (mitogens, osmotic stress, heat shock and pro-inflammatory cytokines) and regulate various cellular activities such as gene expression, mitosis, differentiation, proliferation and cell survival/apoptosis [9,10]. To identify the molecular target of JSI-124 treatment in I-83 (CLL-like B cell line), BJAB (Burkitt lymphoma cell line) and NALM-6 (human pre-acute lymphocytic leukemia cell line), we examined the effect of JSI-124 treatment on MAPK activation. We used these cell lines because they were from B cell derived diseases. Cells were treated with 1 μM JSI-124 or DMSO as vehicle control over a 24 hour time course. Cell extracts were immunoblotted with antibodies against the phosphorylated forms of MAPKs' Erk1/2, p38 and JNK. This was correlated with total protein levels. There was little change observed in phosphorylated p38, and Erk1/2 in BJAB cells whereas there was an increase in p38 and Erk1/2 phosphorylation in I-83 and NALM-6 cells (Figure 1A). In contrast, there was a significant increased in phosphorylation of JNK in a time-dependent fashion in all three B cell derived lines. The phosphorylation levels peaked at 6 hours following JSI-124 treatment and then decreased over time until they were barely detectable at the 24 hours (Figure 1B.i). JNK is recognized as a physiologically important activator of the c-Jun transcription factor [23,24]. Therefore, we measured the phosphorylation and protein levels of c-Jun. At 6 hours of JSI-124 treatment, there was a significant increase in phosphorylation and total c-Jun protein levels in all three cell lines. This was reduced at after 24 hours treatment (Figure 1B.i). In addition, primary cells from patient with CLL were analyzed by immunoblotting with antibodies against p-JNK/JNK and p-c-Jun/c-Jun. Consistent with the results observed in cell lines, p-JNK and p-c-Jun and total c-Jun levels were significantly increased at the 6 hours' time point with JSI-124 treatment and reduced over time (Figure 1B.ii). No change was observed in total JNK level. We further determined phosphorylation status of JNK and c-Jun at earlier time points following JSI-124 treatment. We found no significant change in total JNK protein levels at these earlier time points, however a significant increase in phosphorylation of JNK was observed one hour after treatment with JSI-124. Furthermore, a significant increase in the c-Jun protein level was observed 3 hours after JSI-124 treatment in all three cell lines (Figure 1C). This implies that activation of JNK by JSI-124 also activates c-Jun in these cells. We have previously demonstrated that treatment with 1 μM JSI-124 induced apoptosis and cell cycle arrest at G2 phase in BJAB, I-83, and NALM-6 cells [7]. At this dose, we also observed JNK and c-Jun activation (Figure 1B); therefore we investigated whether c-Jun activation occurs at different dose of JSI-124. I-83, BJAB and NALM-6 cells were treated with various doses of JSI-124 for 6 hours. The results indicated that at a dose as low as 200 nM, JSI-124 induced activation of c-Jun in all three cell lines (Figure 1D). However, in BJAB cells the highest activation of c-Jun was observed at the 0.5 uM concentration. This discrepancy in the activation of c-Jun in BJAB cells might be due to these cells being more sensitive to JSI-124 induced cell death compared to I-83 and NALM-6 cells after treatment. However, even after 24 hours, this lower drug dose did not cause significant apoptosis, by flow cytometric analysis for accumulation of sub-G1 phase and Annexin V staining (Figure 1E). This indicates that JSI-124 activation of the JNK/c-Jun pathway occurs at non-toxic JSI-124 concentrations.

### JSI-124 induced apoptosis and cell cycle arrest was not dependent on activation of JNK/c-Jun in B cell leukemia and lymphoma cells

**Figure 2:**
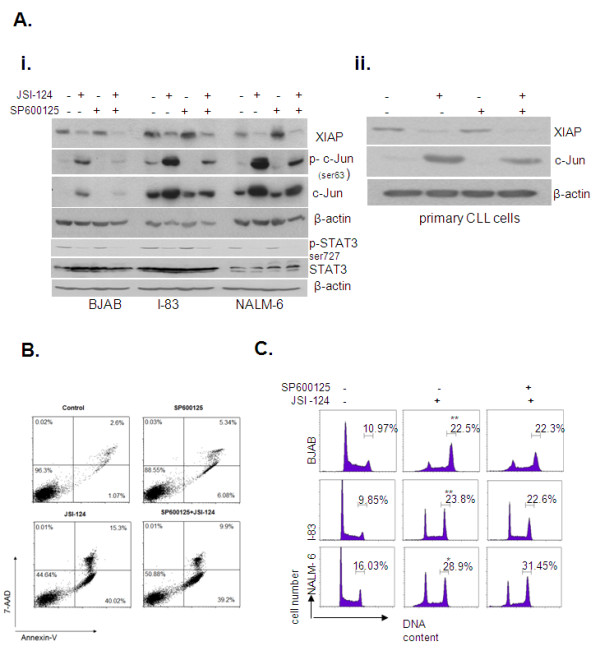
**JSI-124-induced apoptosis was not mediated by c-Jun activation in B-cell malignancies**. **A**. BJAB, I-83, and NALM-6 cells were pre-treated with 20 μM SP600125 followed by 1 μM JSI-124. Decreased levels of phospho-and total c-Jun protein levels were detected by western blotting in the cells treated with the combination of SP600125 and JSI-124 compared to SP600125 alone. Representative original data from three independent experiments are shown. The XIAP and STAT3proteins level, as determined by immunoblotting with antibodies against XIAP and serine phosphor-STAT3/STAT3, did not change with this treatment **B**. Apoptotic cell population was measured using flow cytometric analysis for AnnexinV/PI staining for apoptosis after 24 hour treatment with JSI-124, either alone or in combination with SP600125. DMSO-treated cells were taken as the control. Cells that were 7-AAD-negative and Annexin V-positive were undergoing apoptosis. The percentage of cells in each quadrant is indicated in the quadrant. Representative original data are shown. **C**. Cells were treated with same as above and were analyzed for cell cycle by FACS as described in Materials and Methods. Experiments were done at least three times and representative original data are shown (* represents significant difference of p value of < 0.05 between JSI-124 treated and untreated cells and ** represents a p value of < 0.01).

We have previously demonstrated that JSI-124 induced apoptosis and cycle arrest in B-cell leukemia cells [7]. To investigate the role of the JNK/c-Jun pathway on apoptosis and cell cycle arrest induced by JSI-124, we pretreated I-83, BJAB, NALM-6 and primary CLL cells with the JNK inhibitor SP600125 for one hour followed by JSI-124 treatment for 24 hours. Cell lysates were western blotted for phosphorylated and total c-Jun protein levels. We found that SP600125 treatment led to decreased expression of c-Jun protein in all three cell lines as well as primary CLL cells (Figure 2A i and ii). In contrast, p38 inhibitor SB230850 and Erk1/2 inhibitor U0126, did not effect c-Jun phosphorylation or total protein levels following JSI-124 treatment (additional file 1i and ii). Ubiquitin/proteasome system affects various signaling pathways including JNK/c-Jun pathway [25]. To determine whether JSI-124 mediated c-Jun activation is involved in the proteasome ubiquitin/degradation system, cells were pretreated with MG132, a proteasome inhibitor, followed by JSI-124 treatment. Although MG132 itself induced c-Jun activation in all three cells, no differences in c-Jun protein levels were found between JSI-124 alone or in combination with MG132 treatment (additional file 1i). This data suggests that a proteasome degradation mechanism may not be involved in JSI-124 induced c-Jun activation. When apoptosis was determined by AnnexinV/7AAD staining, JSI-124 induced apoptosis in NALM-6 cells by 1% to 40% (Figure 2B). In addition, although SP600125 treatment dramatically reduced c-Jun protein levels in these cells (Figure 2Ai and ii), SP600125 had no effect on the apoptosis induced by JSI-124 in NALM-6 cells (Figure 2B). Similarly, SP600125 did not influence the degree of apoptosis induced in BJAB and I-83 cells by JSI-124 (data not shown). We and others have shown that JSI-124 induced apoptosis is dependent on down-regulation of XIAP, a member of the IAP (inhibitor of apoptosis protein) family and serine 727 phosphorylation of STAT3 in B-cell leukemia cells and primary CLL cells [6,7]. However, SP600125 had no effect on the reduction in XIAP and serine 727 phosphorylation of STAT3 levels by JSI-124 but did reduce the effect of JSI-124 on c-Jun expression (Figure 2A). Similarly, while cell cycle arrest was observed in all three cell lines (BJAB, I-83, and NALM-6) following treatment with JSI-124 this was not influenced by prior treatment with SP600125 (Figure 2C). The percentage of BJAB cells in G2/M phase increased from 10% to 22% with JSI-124 treatment alone and increased to 22% when the cells were pretreated with SP600125. In I-83 cells, the G2/M fraction increased from 9% to 23% by JSI-124 and 22% by the SP600125 and JSI-124 combination (Figure 2C). Similarly, JSI-124 also induced NALM-6 cell accumulation in G2/M phase from 16% to 29% and no protection was observed by SP600125 (31%) (Figure 2C). These results demonstrate that the effects of JSI-124 on cell growth are unrelated to its effects on the JNK/c-Jun pathway.

**Figure 3:**
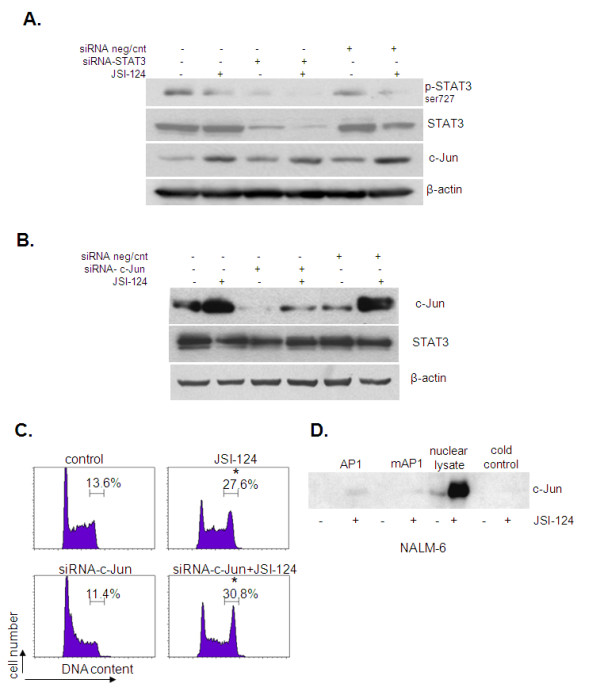
**JSI-124 mediated c-Jun activation was not dependent on STAT3 expression in B-cell leukemia**. **A **and **B**, Cells were transfected with siRNA-STAT or siRNA-c-Jun or siRNA non-target control. Then cells were treated with JSI-124 for additional 6 h. Cell extract was assayed for Western blotting for STAT3 and c-Jun. Knock-down of STAT3 or c-Jun was confirmed by western blotting with antibody to STAT3 or c-Jun. **C**, The same transfected cells were treated with JSI-124 for an additional 24 hours and then cells were analyzed for cell cycle analysis for G1 and G2 phase fractions in the cells transfected with siRNA-c-Jun or non-targeting control siRNA. Standard error was determined on the basis of three independent experiments and an asterisk represents significant difference (a p value of < 0.05) between JSI-124 treated and untreated controls. **D**, Cells were stimulated with JSI-124 and nuclear extracts incubated with the AP1 probe. Pulled down with streptavidin beads, the bound proteins were detected by SDS/polyacrylamide gel electrophoresis and western blotting. To control for specificity, a biotin-labeled scrambled probe was used along with no probe beads control. Blots were probed with antibody to c-Jun.

STAT3 is a transcription factor aberrantly activated in many human solid and hematological cancers and plays a role in oncogenesis [26]. We have previously demonstrated that JSI-124 induces cell cycle arrest at the G2/M phase through inhibition of STAT3 activity in human B leukemic cells [7]. We evaluated whether JSI-124 induced c-Jun activity is dependent on STAT3 expression in the cells. To this end, STAT3 was knocked-down using siRNA before treatment with JSI-124 and the cell lysates were examined for JSI-124 induction of c-Jun by immunoblot analysis in transfected and non-transfected cells. The c-Jun protein level in cells transfected with siRNA against STAT3 or non-targeting control siRNA was same as JSI-124 treated cells (Figure 3A). To control for transfection efficiency, the STAT3 level was assessed in transfected and non-transfected cells and this confirmed a significant decrease in STAT3 in siRNA-treated cells (Figure 3A). Conversely, the STAT3 level was unchanged in cells treated with siRNA against c-Jun (Figure 3B). These data imply that JSI-124-induced activation of c-Jun was not dependent on STAT3 expression in these cells. To determine whether JSI-124induced cell cycle arrest involves activation of c-Jun, I-83 cells were treated with siRNA against c-Jun following JSI-124 treatment and cells were examined for accumulation at G2/M. In I-83 cells, the G2/M phase fraction increased from 13% (untreated) to 27% (JSI-124 treated) and in siRNA treated cells the G2/M phase fraction increased from 11% (siRNA-c-Jun) to 30% (siRNA-c-Jun and JSI-124), respectively Figure 3C. This indicates that the anti-proliferative effect of JSI-124 is independent of c-Jun activation.

Since our results indicated that JSI-124 treatment induced c-Jun activation through JNK phosphorylation, we investigated whether the JNK/c-Jun pathway affects gene transcription. To this end, we isolated the nucleus of NALM-6 cells treated with JSI-124. The nuclear lysates were then incubated with probes containing the consensus DNA binding site for AP-1 or a mutated form of the AP-1 site, and pulled down with beads as described in the Materials and Methods. The beads were then western blotted for the presence of c-Jun. As a control, 10 μg of nuclear lysate from cells that were untreated and treated with JSI-124 were western blotted for c-Jun. We found that c-Jun binds to the AP-1 DNA binding site following JSI-124 treatment whereas c-Jun failed to bind to the mutant AP-1 site (Figure 3D). In addition, untreated nuclear lysate contained a low levels of c-Jun but after JSI-124 treatment the c-Jun levels increased (Figure 3D). This indicates that the JSI-124 treatment increases c-Jun transcriptional activation.

### JSI-124 induced VEGF expression through JNK/c-Jun activation

**Figure 4:**
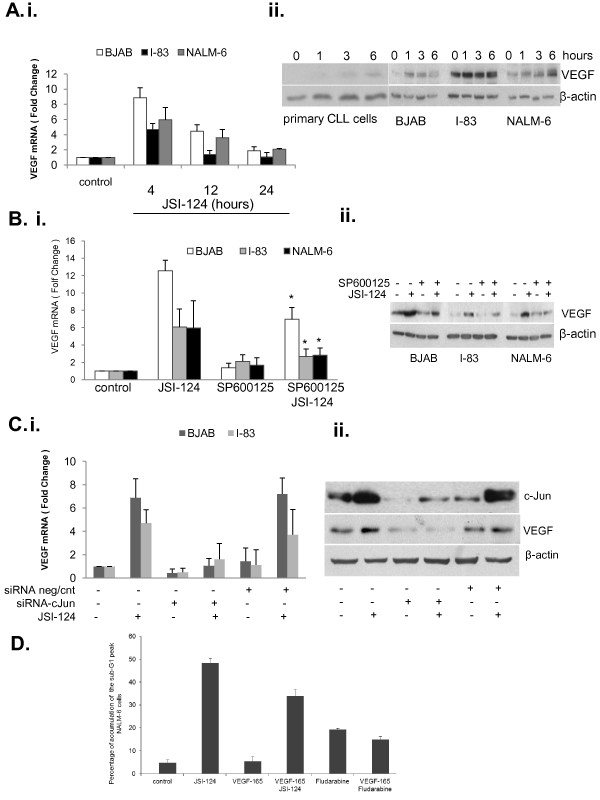
**JSI-124 induced VEGF via activation of c-Jun**. **A**. Increased levels of VEGF protein were detected by western blotting and mRNA by real time PCR in I-83, BJAB, NALM-6 cells after treatment with JSI-124 for the indicated times. Representative original data from four independent experiments are shown. **B**. BJAB, I-83 and NALM-6 cells were pretreated with 20 μmole/ml SP600125 for 1 hour following JSI-124 treatment for additional 4 hours. VEGF mRNA and protein levels were detected by RT-PCR and western blotting, respectively. Standard error was determined on the basis of three independent experiments and an *asterisk *represents significant difference (a *p *value of < 0.05) between JSI-124 alone or pretreated with SP600125. **C**. in BJAB and I-83 cells c-Jun was knocked down by using siRNA for c-Jun or non-target control siRNA. Total RNA was extracted and real time-PCR was used to detect mRNA level of VEGF. Error bars represent the standard errors for three independent experiments. Cell extract was assayed for western blotting for VEGF. Knock-down of c-Jun was confirmed by western blotting with antibody to c-Jun. **D**. NALM-6 cells were treated with 20 ng/ml VEGF-165 for 1 h and followed by JSI-124 for 24 h. The percentages of apoptotic cells were determined by FACS analysis for sub-G1 accumulation.

One of the genes up-regulated by the JNK signaling pathway is VEGF [17,26,27]. In Figure 4A.i, VEGF mRNA increased at 4 hours in BJAB, I-83 and NALM-6 cells treated with JSI-124 and the levels then declined over a 24 hour time course. VEGF protein levels in JSI-124 treated cells increased in parallel with the VEGF mRNA levels (Figure 4A.ii). A small increase in VEGF protein levels was observed in primary CLL cells treated with JSI-124 (Figure 4Aiii). To determine the relative induction of VEGF expression compared to other stress response genes, we treated BJAB cells with JSI-124 for 6 hours and performed super array analysis with mRNA isolated from JSI-124 treated and control cells using a kit from Human Signal Transduction PathwayFinder™ RT^2^*Profiler*™ (SABiosciences, QIAGEN). Consistent with our findings above, there was an 8.7-fold increase in c-Jun and 13.2 fold increase in VEGF-A mRNA level in cells treated with JSI-124 (additional file 2). Furthermore, we also observed a 4.99-fold increase in c-Fos mRNA level (additional file 2). This indicates that JSI-124 induces VEGF expression in B leukemic cell lines.

The requirement for the JNK/c-Jun pathway to increase VEGF expression was also evaluated. BJAB, I-83 and NALM-6 cells were pretreated with the JNK inhibitor, SP600125 followed by JSI-124 treatment. We found that the pretreatment with SP600125 significantly blocked JSI-124-induced VEGF mRNA (Figure 4Bi) and protein (Figure 4Bii) increases in these cells. In addition, the ability of c-Jun to regulate JNK-induced VEGF expression was also investigated. We have determined that SP600125 blocked c-Jun activation in B leukemia cell lines (Figure 2A). This indicates that activation of c-Jun may regulate VEGF expression in these cells. To confirm the involvement of c-Jun in JSI-124 induced VEGF expression, c-Jun was knocked-down using siRNA and cell lysates were examined for VEGF mRNA expression. JSI-124 treatment failed to induce VEGF mRNA in the presence of siRNA against c-Jun whereas the VEGF mRNA level was increased in cells transfected with non-targeting control siRNA (Figure 4C.i). The transfection efficiency of siRNA against c-Jun was assessed by immunoblot analysis of c-Jun expression in transfected and non-transfected cells. This showed a significant decrease in c-Jun protein levels in cell transfected with siRNA against c-Jun. β-actin expression was used as the loading control (Figure 4C.ii). Moreover, VEGF protein levels, which were increased in JSI-124 treated cells, were significantly decreased in cells transfected with siRNA against c-Jun (Figure 4C.ii). Cells transfected with non-targeting control siRNA showed same amount of VEGF protein as non-transfected cells (Figure 4C.ii). Next, we studied whether VEGF could block JSI-124 induced apoptosis. NALM-6 cells were pretreated with VEGF for one hour followed by JSI-124 treatment for 24 h. We observed that JSI-124 induced apoptosis was reduced by ~25% with VEGF pretreatment (Figure 4D). Taken together, these results indicate that JSI-124 induced VEGF expression through activation of JNK/c-Jun pathway in B derived leukemia cells.

## Discussion

The antitumor activity of JSI-124 has been attributed to its effects on STAT3 signaling [1,2]. This inhibitor has been shown to exert anti-proliferative and anti-survival activity both *in vivo *and *in vitro *[1,3,7,26]. This indicates that targeting the STAT3 pathway by JSI-124 could be an effective treatment for these cancers. Although STAT3 is constitutively phosphorylated on serine 727 residue in B CLL cells [7], it is upstream mediator is not known. MAPKs Erk, but not JNK and p38, phosphorylate serine 727 of STAT3 *in vitro *and *in vivo *[28]. However, other study demonstrated that MEKK7 mediated JNK1 phosphorylate serine 727 of STAT3 [29,30]. We found no change in serine phospho-STAT3 levels in cells treated with JNK inhibitor SP600125 alone or in combination with JSI-124. Therefore, the activation of JNK by JSI-124 is independent of JSI-124 inhibition of STAT3 function in B-cell lines. These results could be due to the differential activation of JNKs, different types of stimuli and/or different cell types. Furthermore, we demonstrate that JSI-124 activates the JNK/c-Jun signaling pathway and this lead to increased VEGF expression. Although inhibition of JNK/c-Jun reduced expression of VEGF induced by JSI-124, we failed to detect an increase in apoptosis in the B-cell leukemia and lymphoma cells and using VEGF receptor inhibitor also failed to increase JSI-124 induced apoptosis (data not shown). Even though JNK or VEGF receptor inhibition failed to block JSI-124 induced apoptosis, JSI-124 mediated VEGF secretion and accumulation in microenvironments might contribute to drug resistance. Indeed, VEGF signaling has important implication for apoptosis resistance, and enhanced survival of B CLL cells [21]. CLL cells spontaneously secrete VEGF and induce autocrine signaling pathway in the same cells [31]. As STAT3 has been shown to interact with VEGF-R1/2 in CLL [32], it is well characterized that VEGF activation is associated with Jak-tyrosine kinase activation [12-16]. For example, in T cell lymphoma, VEGF upregulation was associated with activation of Jak-tyrosine kinase and JNKs activation but was independent of STAT3 activity [33]. This study demonstrates that JSI-124 activates the JNK/c-Jun pathway independent of STAT3 in B leukemic cells.

JNK was originally identified as a kinase that binds and phosphorylates c-Jun on Ser63 and Ser73 within its transcriptional activation domain [9,10]. JNK is activated in response to various stress stimuli such as environmental stress, including UV, osmotic shock, inflammatory cytokines and chemotherapic drugs [9,10,34]. The studies using JNK knockout mice suggested its important role in leukemia and skin tumorigenesis and insulin resistant diabetes [34,35]. JNK phosphorylates diverse substrates but one important function is the ability to phosphorylate c-Jun and induce AP-1 dependent transcription [10,34]. When phosphorylated, c-Jun forms either monodimer or heterodimer with c-Fos. The heterodimer c-Jun/c-Fos binds to the AP-1 DNA binding sites more efficiently that the c-Jun monodimer does [10,34]. In this study we found that JSI-124 induced JNK mediated c-Jun phosphorylation and its transcriptional activation for binding to AP-1-DNA site. In addition, we also observed that JSI-124 induced activation of Fos mRNA. Therefore we speculate that JSI-124 might be inducing heterodimer formation of c-Jun/c-Fos. This will be elucidated in further studies. More interestingly, we also observed that even nontoxic dose of JSI-124 caused constitutive activation of c-Jun suggesting JSI-124 activation of the JNK/c-Jun pathway is one of the earliest responses to JSI-124 treatments in these cells.

It is known that an important factor involved in VEGF induction is JNK signaling [36,37] and JNK induces c-Jun phosphorylation at the VEGF promoter [37]. Genetic or pharmacological inhibition of JNK/c-Jun reduces VEGF expression. In detail, we found that JSI-124 selectively up-regulated VEGF expression in response to acute exposure (3-6 hours) and this was inhibited by JNK inhibitor SP600125 or siRNA against c-Jun in B cell tumors. Consistent with our finding, it was shown before that JNKs induce VEGF expression by increasing c-Jun/AP1 activity in T-cell lymphomas [33].

On the other hand, VEGF has been found to be one of the key regulators of angiogenesis in many cancers, including chronic lymphocytic leukemia [21,27,38]. Previously we have shown that regulation of VEGF expression is controlled by JNK and NFκB activation in BJAB cells [21]. This leads to activation of VEGF receptors and cell survival in B-cell derived malignancies including CLL. Genotoxic stress has been found to induce VEGF expression. For example, human melanoma cells treated with the antineoplastic drug, dacarbazine, produces an increase in secreted VEGF [39]. Also, ultraviolet irradiation or photodynamic therapy can increase tumor cell VEGF secretion from keratinocytes or prostate cancer cells respectively [40,41]. It has also been shown that some tumors derived from c-Jun-transformed cells exhibit a higher level of formation of functional microvascular networks than those derived from other oncogenes [42].

## Consclusions

Our study indicates that, in contrast to its inhibition of STAT3, JSI-124 activates the JNK signaling pathway leading to VEGF expression. This suggests that JSI-124 is inducing a stress response in B leukemia cells potentially leading to increased angiogenesis. Future studies will be aimed to understand whether inhibition of VEGF may be targeted therapeutically to enhance JSI-124-induced cell death in CLL and B-cell malignancy. Taken together, our study provides new insight into the molecular effects of this potentially important new chemotherapeutic agent.

## Competing interests

The authors declare that they have no competing interests.

## Authors' contributions

GI carried out the experiments and drafted the manuscript. JBJ participated in the collection of patients' samples and in the design and coordination of the study. SBG conceived of the study, and participated in its design and coordination. All authors read and approved the final manuscript.

## Pre-publication history

The pre-publication history for this paper can be accessed here:

http://www.biomedcentral.com/1471-2407/11/268/prepub

## Supplementary Material

Additional file 1 **p38 and Erk1/2 signaling was not involved in JSI-124 mediated c-Jun activation**. BJAB, I-83, and NALM-6 cells were pretreated with 20 μM SP230850 or U0126 or 5 uM MG132 following 1 μM JSI-124 treatment. c-Jun protein levels were detected by western blotting Representative original data from three independent experiments are shown. Click here for file

Additional file 2 **Supper Array analysis**. BJAB cell were treated by JSI-124 for 6 hours. Total RNA was extracted as described in Materials and Methods. 84 genes Super Array analysis was carried out by using kit for Human Signal Transduction PathwayFinder™ RT^2^*Profiler*™ Human Signal Transduction PathwayFinder™ RT^2^*Profiler*™ (SABiosciences). Click here for file
