# Supplementary material for: Cucurbitacin-I (JSI-124) activates the JNK/c-Jun signaling pathway independent of apoptosis and cell cycle arrest in B Leukemic Cells
Source: BMC Cancer. 2011 Jun 24;11:268. doi: 10.1186/1471-2407-11-268 (PMC3146936; doi:10.1186/1471-2407-11-268)
Supplement: Additional file 1 — p38 and Erk1/2 signaling was not involved in JSI-124 mediated c-Jun activation. BJAB, I-83, and NALM-6 cells were pretreated with 20 μM SP230850 or U0126 or 5 uM MG132 following 1 μM JSI-124 treatment. c-Jun protein levels were detected by western blotting Representative original data from three independent experiments are shown. [file 1471-2407-11-268-S1.PPT]

## Slide 1
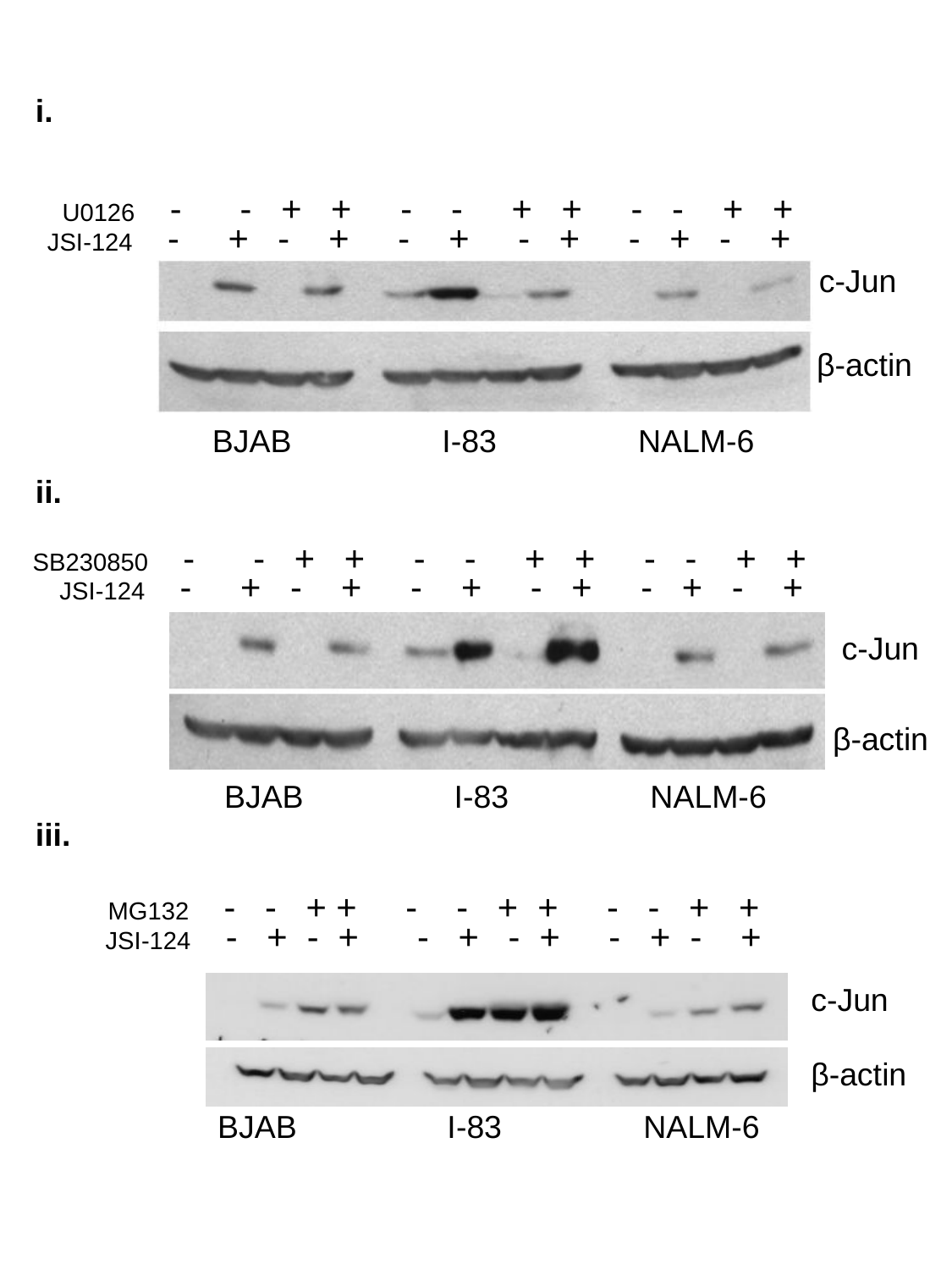

i.
ii.
iii.
 U0126 - - + + - - + + - - + +
JSI-124 - + - + - + - + - + - +
c-Jun
β-actin
BJAB I-83 NALM-6
SB230850 - - + + - - + + - - + +
JSI-124 - + - + - + - + - + - +
c-Jun
β-actin
BJAB I-83 NALM-6
MG132 - - + + - - + + - - + +
JSI-124 - + - + - + - + - + - +
c-Jun
β-actin
BJAB I-83 NALM-6
