# Supplementary figures and images for: Cucurbitacin-I (JSI-124) activates the JNK/c-Jun signaling pathway independent of apoptosis and cell cycle arrest in B Leukemic Cells
Source: BMC Cancer. 2011 Jun 24;11:268. doi: 10.1186/1471-2407-11-268 (PMC3146936; doi:10.1186/1471-2407-11-268)

## Slide 1
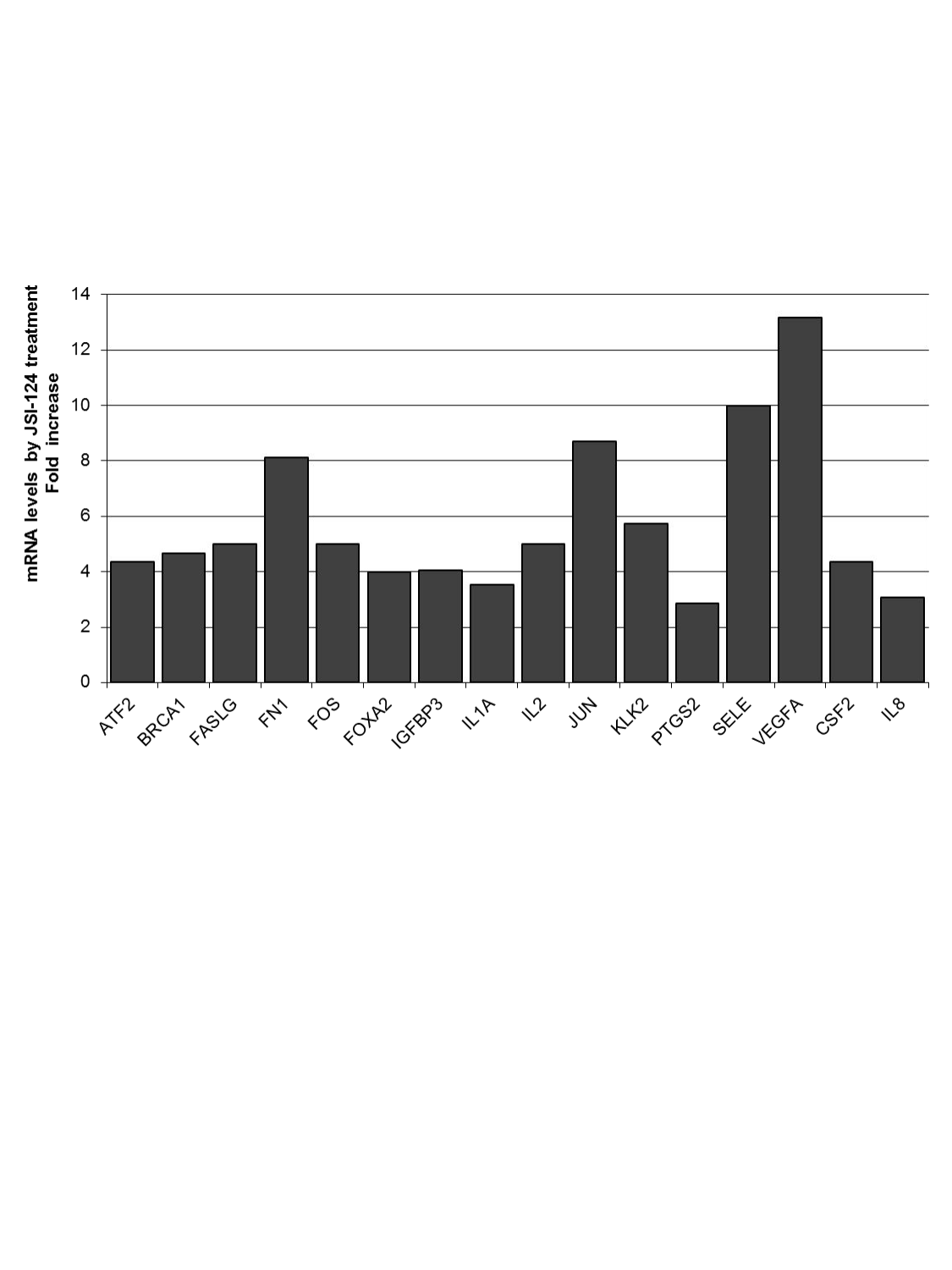

Supplement: Additional file 2 — Supper Array analysis. BJAB cell were treated by JSI-124 for 6 hours. Total RNA was extracted as described in Materials and Methods. 84 genes Super Array analysis was carried out by using kit for Human Signal Transduction PathwayFinder™ RT2Profiler™ Human Signal Transduction PathwayFinder™ RT2Profiler™ (SABiosciences). [file 1471-2407-11-268-S2.PPT]
